# Racial and microvascular determinants of progression to treatment-warranted diabetic eye disease

**DOI:** 10.1038/s41433-026-04417-0

**Published:** 2026-04-07

**Authors:** Alexander T. Hong, Ivan Y. Luu, Tze-Woei Tan, Brian C. Toy

**Affiliations:** 1 Roski Eye Institute, Keck School of Medicine, University of Southern California, Los Angeles, CA USA; 2 Division of Vascular Surgery and Endovascular Therapy, Keck School of Medicine, University of Southern California, Los Angeles, CA USA

**Keywords:** Retinal diseases, Metabolic disorders, Risk factors, Outcomes research

## Abstract

**Objective:**

To evaluate racial and ethnic disparities in progression from non-proliferative diabetic retinopathy (NPDR) to treatment-warranted diabetic eye disease (TW-DED), and the influence of microvascular diabetic complications, including nephropathy (DN) and foot ulcers (DFU).

**Methods:**

We conducted a retrospective cohort study using a federated electronic health records network (2005–2025) of adults ≥40 years with type 2 diabetes and NPDR, excluding those with prior TW-DED. Participants were stratified by race/ethnicity (White, Hispanic, Black, Asian, Other) and by DN and/or DFU status. Propensity score matching (1:1) balanced baseline characteristics. The primary outcome was progression to TW-DED, defined as proliferative diabetic retinopathy, macular oedema, vitreous haemorrhage, or need for retinopathy-related treatment, assessed over 10 years. Risk ratios (RR) and hazard ratios (HR) with 95% confidence intervals (CI) were reported.

**Results:**

Among 130,002 patients with NPDR, Hispanic (RR 1.40, 95% CI 1.32–1.48), Black (RR 1.15, 95% CI 1.10–1.20), and Other (RR 1.13, 95% CI 1.04–1.24) patients demonstrated higher risks of TW-DED progression than White patients. In multivariable analysis, race/ethnicity was no longer significant, whereas DFU (HR 1.08, 95% CI 1.02–1.15) and DN (HR 1.06, 95% CI 1.01–1.12) remained independently associated with TW-DED. Stratified analysis revealed DFU consistently conferred greater risk than DN (Hispanic HR 1.39; Other HR 1.32; Black HR 1.27; White HR 1.22).

**Conclusion:**

Hispanic, Black, and Other groups had higher risk of TW-DED progression, but differences diminished after comorbidity adjustment. DFU and DN independently predicted progression, with DFU posing greater risk, suggesting microvascular disease burden and management differences may underlie racial differences.

## Introduction

Diabetic retinopathy (DR) is a common microvascular complication of type 2 diabetes mellitus (T2DM) and a major contributor to preventable vision loss, affecting nearly one-third of individuals with T2DM worldwide [1]. Advanced stages, particularly proliferative diabetic retinopathy (PDR) and diabetic macular oedema (DME), are responsible for considerable morbidity and increasing healthcare system burden [2, 3]. Chronic hyperglycaemia drives DR progression through inflammatory mediator activation and ischaemic injury [4]. Other diabetic microvascular complications, including diabetic nephropathy (DN) and diabetic foot ulcers (DFU), share common risk factors and pathophysiologic pathways, yet the interaction between these microvascular complications and their contribution to diabetic eye disease remains understudied [5, 6].

A growing body of research suggests that racial and ethnic differences contribute significantly to disparities in diabetic complications [7, 8]. Minority populations, including Black and Hispanic individuals, are more likely to present with advanced DR, require invasive interventions, and experience worse visual outcomes compared with their White counterparts [8, 9]. Factors contributing to these differences include delayed access to care, differences in healthcare utilisation, socioeconomic barriers, and variations in disease severity at presentation [10, 11]. However, studies rarely consider the influence of coexisting microvascular complications like DN and DFU, conditions that may reflect more advanced systemic disease, on DR progression across racial and ethnic groups [12, 13]. This gap in understanding limits the development of targeted strategies to prevent vision-threatening complications and address systemic inequities in diabetes care.

While research has documented racial differences in DR prevalence and progression, there is a lack of large-scale, longitudinal data assessing how microvascular comorbidities contribute to progression to treatment-warranted diabetic eye disease (TW-DED), particularly across diverse racial and ethnic groups [9, 14]. The extent to which race modifies the impact of DN and DFU on DR outcomes remains unclear, as structural barriers may also disproportionately affect minority populations with these comorbidities [15, 16]. Our study aims to fill these critical knowledge gaps by analysing a national, diverse cohort of individuals with type 2 diabetes to investigate racial and ethnic disparities in the progression to TW-DED, with a focus on how DFU and DN influence these risks.

## Methods

### Data source and study population

This retrospective cohort study was conducted using the United States (U.S.) Collaborative Network within the TriNetX Analytics platform, a large federated electronic health records network that compiles deidentified electronic health record (EHR) data from over 118 million patients across 69 U.S. healthcare organisations (HCO). The participating institutions include both academic and community-based centres, as well as their affiliated sites, and encompass data from insured and uninsured patients [17]. Due to the use of deidentified data, the study was deemed exempt by the University of Southern California institutional review board, and informed consent was not required. All procedures complied with the Health Insurance Portability and Accountability Act, tenets of the Declaration of Helsinki, and Strengthening the Reporting of Observational Studies in Epidemiology reporting guidelines for cohort studies [18]. All data were collected in June 2025, with the study period spanning from June 2005 to June 2025.

Study individuals (≥40 years old) were identified using International Classification of Diseases, Tenth Revision (ICD-10) codes for type 2 diabetes with non-proliferative diabetic retinopathy (NPDR) (E11.32-E11.34). TW-DED was defined as the development of PDR, vitreous haemorrhage (VH), DME, or receipt of related treatments for DR, including intravitreal injection (IVI), panretinal or focal photocoagulation (PRP), and pars plana vitrectomy (PPV). Patients with any diagnosis of TW-DED or its components before the first recorded NPDR diagnosis were excluded. The index event was the date of initial NPDR diagnosis.

The NPDR cohort was stratified by race and ethnicity groups as recorded in the HER [19]. These five groups included: 1) Non-Hispanic White (White), 2) Hispanic, 3) Non-Hispanic African American or Black (Black), 4) Non-Hispanic Asian (Asian), 5) Other (Other race, Native American or Pacific Islander, or American Indian or Alaska Native). The NPDR cohort was also stratified by DFU (ICD-10 L97) and DN (ICD-10 E11.21) status at baseline [20, 21]. Codes used in analysis can be found in Supplementay Table 1.

### Propensity score matching

Propensity score matching was conducted across race-stratified NPDR cohorts (Supplementary Tables 2–5) using 1:1 greedy nearest-neighbour matching with a calliper of 0.1 pooled standard deviations of the logit of the propensity score. Only available patient data were used; missing values were not imputed, and variables with missing data were excluded unless values were present. Covariate balance after matching was assessed by standardised mean differences (SMD), with SMD < 0.10 indicating acceptable balance. Variables included in matching were age at index, sex (as defined in the EMR), comorbidities, socioeconomic status and psychosocial health hazards, tobacco use, insulin, oral diabetic agents, antihypertensive agents, antithrombotic agents, body mass index (BMI), glycosylated haemoglobin (HbA1c), cholesterol, glomerular filtration rate (GFR), triglycerides, and healthcare utilisation (outpatient, inpatient, and emergency department visits).

### Exposure and outcomes

Exposures included race/ethnicity as well as presence of DFU (ICD-10: L97.xx) and DN (ICD-10: E11.2x) at baseline. Codes for DFU [22, 23] and DN [24, 25] were derived from previous validated studies. The primary outcome was progression to TW-DED, while secondary outcomes included the separate components of TW-DED (PDR, VH, DME, IVI, PRP, and PPV) and progression to blindness or low vision. Outcomes were assessed at 1-, 5-, and 10-year intervals after the index event.

### Statistical analysis

Analyses were conducted using the native analytic functions of the TriNetX platform. Descriptive statistics summarised baseline characteristics, with continuous measures expressed as mean ± standard deviation (SD) and categorical variables as proportions and percentages (%). Statistical significance was set at two-sided *P*-value < 0.05. Our primary analysis determined whether risk of progression to TW-DED disproportionately affected certain racial/ethnic groups, and multiple pairwise comparisons were conducted with non-Hispanic White individuals set as the reference group (Table 1). Risk ratios (RRs) were quantified for the development of TW-DED and individual outcomes, calculated as the proportion of matched individuals with the outcome in the exposed group (e.g., matched Hispanic with TW-DED / matched Hispanic) divided by the proportion in the reference group (e.g., matched White with TW-DED / matched White), with 95% confidence intervals (CIs) reported for all outcomes.

**Table 1:** Risk of progression to treatment-warranted diabetic eye disease among patients with non-proliferative diabetic retinopathy stratified by ethnic cohorts compared to non-Hispanic White individuals.

	1 year	5 years	10 years
Outcome	RR (95% CI)	RR (95% CI)	RR (95% CI)
*Hispanic*			
TW-DED	1.50 (1.38, 1.64)	1.35 (1.27, 1.42)	1.40 (1.32, 1.48)
PDR	1.87 (1.64, 2.14)	1.78 (1.62, 1.95)	1.65 (1.51, 1.82)
VH	1.67 (1.17, 2.38)	1.82 (1.49, 2.23)	1.45 (1.08, 1.94)
DMO	1.51 (1.35, 1.69)	1.35 (1.25, 1.45)	1.45 (1.35, 1.56)
IVI	1.75 (1.46, 2.10)	1.61 (1.42, 1.82)	1.35 (1.20, 1.53)
PRP	2.31 (1.90, 2.82)	2.41 (2.19, 2.77)	1.88 (1.63, 2.16)
PPV	1.68 (1.26, 2.24)	1.71 (1.41, 2.10)	1.82 (1.49, 2.22)
Blindness or low vision	1.01 (0.80, 1.28)	1.16 (1.04, 1.30)	1.37 (1.23, 1.53)
*Black*			
TW-DED	1.18 (1.10, 1.27)	1.15 (1.09, 1.20)	1.15 (1.10, 1.20)
PDR	1.24 (1.10, 1.40)	1.29 (1.19, 1.40)	1.29 (1.20, 1.39)
VH	1.07 (0.78, 1.46)	1.07 (0.89, 1.30)	1.05 (0.89, 1.24)
DMO	1.22 (1.12, 1.34)	1.12 (1.06, 1.19)	1.16 (1.10, 1.23)
IVI	1.11 (0.95, 1.30)	1.17 (1.05, 1.31)	1.11 (1.01, 1.23)
PRP	1.40 (1.16, 1.70)	1.38 (1.22, 1.57)	1.38 (1.23, 1.56)
PPV	0.93 (0.71, 1.21)	1.18 (0.98, 1.42)	1.03 (0.88, 1.21)
Blindness or low vision	0.93 (0.83, 1.04)	1.24 (1.14, 1.35)	1.12 (1.04, 1.21)
*Asian*			
TW-DED	0.92 (0.80, 1.06)	0.98 (0.89, 1.08)	1.01 (0.93, 1.10)
PDR	1.08 (0.85, 1.38)	1.11 (0.95, 1.30)	1.13 (0.97, 1.30)
VH	0.68 (0.37, 1.26)	1.21 (0.85, 1.71)	1.11 (0.81, 1.51)
DMO	0.85 (0.71, 1.03)	0.88 (0.78, 1.00)	0.93 (0.83, 1.04)
IVI	0.93 (0.69, 1.26)	0.83 (0.67, 1.03)	0.81 (0.66, 0.99)
PRP	1.73 (1.19, 2.50)	1.32 (1.03, 1.71)	1.29 (1.02, 1.63)
PPV	0.89 (0.51, 1.55)	1.09 (0.78, 1.53)	1.00 (0.73, 1.37)
Blindness or low vision	0.82 (0.65, 1.04)	0.98 (0.82, 1.16)	0.98 (0.83, 1.14)
*Other*			
TW-DED	1.23 (1.07, 1.41)	1.15 (1.05, 1.27)	1.13 (1.04, 1.24)
PDR	1.46 (1.16, 1.84)	1.30 (1.12, 1.52)	1.27 (1.10, 1.46)
VH	0.80 (0.42, 1.54)	1.02 (0.71, 1.47)	0.97 (0.71, 1.34)
DMO	1.11 (0.93, 1.34)	1.10 (0.98, 1.25)	1.09 (0.97, 1.22)
IVI	0.99 (0.71, 1.37)	0.92 (0.73, 1.16)	0.89 (0.72, 1.10)
PRP	1.41 (0.95, 2.08)	1.22 (0.93, 1.59)	1.16 (0.90, 1.49)
PPV	1.00 (0.59, 1.69)	1.30 (0.90, 1.88)	1.50 (1.09, 2.07)
Blindness or low vision	0.98 (0.76, 1.27)	1.00 (0.84, 1.19)	0.97 (0.82, 1.14)

Univariable pairwise analysis was conducted using 1:1 propensity score matching to compare the risk of progression to TW-DED among racial and ethnic minority patients with non-proliferative diabetic retinopathy (NPDR), with White patients as the reference group. Matching accounted for clinical characteristics relevant to TW-DED progression. Risk ratios (RRs) with 95% confidence intervals (CIs) were calculated as the proportion of TW-DED cases within each matched racial/ethnic cohort divided by the proportion of cases in the matched White cohort. A RR > 1 indicates increased risk of the outcome in the respective racial/ethnic cohort compared to the White cohort.

*TW-DED* treatment-warranted diabetic eye disease, *RR* risk ratio, *CI* confidence interval, *PDR* proliferative diabetic retinopathy, *DMO* diabetic macular oedema, *VH* vitreous haemorrhage, *IVI* intravitreal injection, *PRP* panretinal photocoagulation, *PPV* pars plana vitrectomy.

We employed three complementary secondary analyses using multivariable Cox proportional hazards models to calculate hazard ratios (HRs) with 95% CIs to assess the impact of DFU and DN on progression to TW-DED. Patients were right-censored at the time of their last documented clinical encounter to account for loss to follow-up. Sub-analyses included 1) race-adjusted multivariable Cox analysis comparing those with and without TW-DED, (Supplementary Table 6), and 2) analyses stratified by race and baseline DFU/DN status (e.g., comparing White individuals with/without DN at baseline to assess the effect of DFU) to evaluate the interaction between race and DFU/DN on progression to TW-DED (Table 2, Supplementary Tables 7, 8).

**Table 2:** Independent associations of diabetic foot ulcers and diabetic neuropathy on treatment-warranted diabetic eye disease progression in patients with non-proliferative diabetic retinopathy, stratified by race/ethnicity.

Race/Ethnicity	Cohort	Covariate	HR (95% CI)
White	DN	DFU	1.22 (1.11, 1.34)
	DFU	DN	1.10 (1.01, 1.21)
Hispanic	DN	DFU	1.39 (1.22, 1.60)
	DFU	DN	1.13 (1.01, 1.28)
Black	DN	DFU	1.27 (1.10, 1.47)
	DFU	DN	1.24 (1.10, 1.41)
Asian	DN	DFU	1.17 (0.78, 1.73)
	DFU	DN	1.02 (0.79, 1.33)
Other	DN	DFU	1.32 (1.03, 1.70)
	DFU	DN	0.97 (0.75, 1.26)

Multivariable Cox proportional hazards models stratified by both race/ethnicity and baseline DFU or DN status. An HR > 1 indicates increased risk of TW-DED after multivariable adjustment. For example, among White individuals with DN at baseline, those with DFU have a higher risk of TW-DED progression than White individuals with DN but without DFU. The full models with all covariates are reported in Supplementary Table 7 (DN cohorts) and Supplementary Table 8 (DFU cohorts).

*HR* hazard ratio, *CI* confidence interval, *DN* diabetic nephropathy, *DFU* diabetic foot ulcer, *NPDR* non-proliferative diabetic retinopathy, *TW-DED* treatment warranted-diabetic eye disease.

## Results

### Cohort demographics

**Fig. 1:**
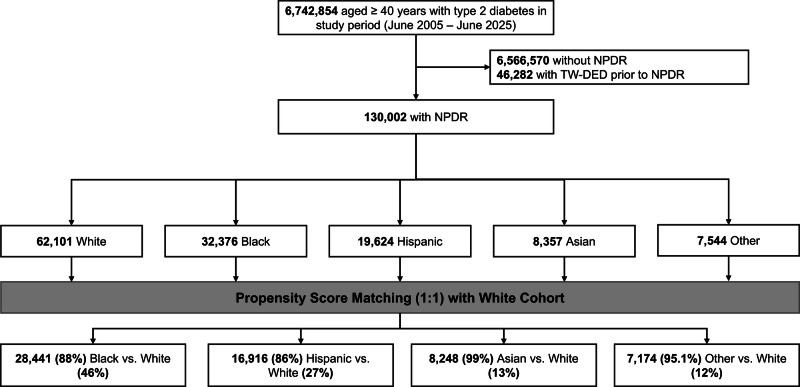
Study flow diagram. Flowchart of adults (≥40 years) with type 2 diabetes and non-proliferative diabetic retinopathy (NPDR), excluding those with prior treatment-warranted diabetic eye disease (TW-DED). NPDR cohorts were stratified by race/ethnicity, and each minority cohort was propensity score matched to the White cohort to assess progression to TW‑DED.

A total of 130,002 patients with NPDR were identified (Fig. 1). The mean age at index was 61.6 ± 12.6 years, with 43% female. The racial/ethnic distribution included 48% White, 25% Black, 15% Hispanic, 6% Asian, and 6% Other. Among comorbid conditions at baseline, 62% had hypertension, 25% had ischaemic heart disease, 35% had DN, and 13% had DFU. Regarding medication use, 55% were on insulin, 68% were taking non-insulin antiglycaemic agents (including metformin), 11% were on glucagon-like peptide-1 receptor agonists (GLP-1 RA), and 73% were using lipid-modifying agents. Patients were followed for a mean ± SD of 4.2 ± 3.0 years.

### Primary analysis

Of those with NPDR, 62,101 were in the White and 19,624 in the Hispanic cohort. After propensity score matching, 16,916 (86.2% of Hispanic) patients remained in each cohort (Supplementary Table 2). Groups were well-matched besides Hispanic cohort showing higher HbA1c (8.5 ± 2.1 vs. 8.3 ± 2.2, SMD = 0.117) and lower BMI (31.4 ± 6.8 vs. 32.4 ± 8.0, SMD = 0.124). Compared to White patients, Hispanic patients showed increased risk of progression to TW-DED at 1- (RR 1.50, 95% CI 1.38–1.64), 5- (RR 1.35, 95% CI 1.27–1.42) and 10-year follow-up (RR 1.40, 95% CI 1.32–1.48) (Table 1). Among components of TW-DED, Hispanic patients showed increased risk for PDR, DME, VH, and use of IVI, PRP, and PPV at 10-years. Risk of blindness or low vision was also increased in the Hispanic cohort at 5- (RR 1.16, 95% CI 1.04–1.30) and 10-year (RR 1.37, 95% CI 1.23–1.53) follow-up.

32,376 Black patients were identified. After propensity score matching with the White cohort, 28,441 (87.8% of Black) patients were harmonised to each cohort, with well-balanced groups (Supplementary Table 3). Compared to White patients, Black patients showed increased risk of progression to TW-DED at 1- (RR 1.18, 95% CI 1.10–1.27), 5- (RR 1.15, 95% CI 1.09–1.20), and 10-year follow-up (RR 1.15, 95% CI 1.10–1.20) (Table 1). Among components of TW-DED, Black patients showed increased risk for PDR, DME, and use of PRP at 10-years. Risk of blindness or low vision was also increased in the Black cohort at 5- (RR 1.24, 95% CI 1.14–1.35) and 10- (RR 1.12, 95% CI 1.04–1.21) year follow-up.

8357 Asian patients were identified. After propensity score matching with the White cohort, 8248 (98.7% of Asian) patients remained in each cohort (Supplementary Table 4). BMI was lower in the Asian cohort (27.4 ± 5.4 vs. 28.6 ± 6.7, SMD = 0.195). Compared to White patients, Asian patients showed no increase in risk of progression to TW-DED at 1- (RR 0.92, 95% CI 0.81–1.06), 5- (RR 0.98, 95% CI 0.89–1.08), and 10-year (RR 1.01, 95% CI 0.93–1.10) follow-up compared to White patients (Table 1). Compared to White patients, Asian patients continued to show increased risk of PRP use at 5- (RR 1.32, 95% CI 1.03–1.71) and 10- (RR 1.29, 95% CI 1.02–1.63) year follow-up. Risk of blindness or low vision was similar between Asian and White cohorts.

7544 patients were identified in the Other cohort. After matching with the White cohort, 7174 (95.1% of Other) patients remained in each cohort (Supplementary Table 5). HbA1c was higher in the Other cohort (8.5 ± 2.2 vs. 8.2 ± 2.2, SMD = 0.143). Compared to the White cohort, the Other cohort showed significantly increased risk of progression to TW-DED at 1- (RR 1.23, 95% CI 1.07–1.41), 5- (RR 1.15, 95% CI 1.05–1.27), and 10-year follow up (RR 1.13, 95% CI 1.04–1.24) (Table 1). Among components of TW-DED, the Other cohort showed increased risk for PDR and PPV at 10-years. Risk of blindness or low vision was similar between Other and White cohorts.

### Association of DFU and DN on TW-DED progression

Subsequently, we assessed the contribution of DFU and DN to TW-DED progression using a multivariable Cox proportional hazards model comparing patients with NPDR who progressed to TW-DED to those who did not (Supplementary Table 6). After adjusting for demographics and comorbidities, race and ethnicity showed non-significance in the multivariable model. Both DFU (HR 1.08, 95% CI 1.02–1.15) and DN (HR 1.06, 95% CI 1.01–1.12) were independently associated with TW-DED progression.

To assess the interactions between race, DFU, and DN on TW-DED progression, stratified analysis revealed that in patients with DN at baseline, the presence of DFU was associated with amplified risk of TW-DED progression across all racial cohorts besides Asian (Table 2, Supplementary Table 7). In patients with DFU at baseline, presence of DN was associated with increased TW-DED risk in White, Hispanic, and Black cohorts, but not Asian and Other (Table 2, Supplementary Table 8).

## Discussion

In this national cohort study of 130,002 patients with NPDR, we observed significant racial and ethnic disparities in the progression to TW-DED. Compared to White individuals, Hispanic, Black, and Other individuals showed an increased risk of progression to TW-DED, with highest rates in Hispanic patients and lowest risk among Asian patients. Elevated risk of TW-DED in Hispanic and Black populations was mirrored by higher rates of blindness/low vision at 5 and 10 years of follow-up. We evaluated individual components of TW-DED among the racial subgroups and found a higher risk of PDR among all non-White groups. Meanwhile, DME risk was higher for Hispanics and Blacks, and VH for Hispanics. Together, these findings suggest that progression to VH and DME may be primary drivers of blindness or low vision among patients with NPDR. Despite greater utilisation of vision-saving interventions (IVI, PRP, and PPV) in Hispanic and Black patients, disparities in vision outcomes persisted, even after adjusting for key microvascular comorbidities. Furthermore, stratification by DFU and DN status revealed that presence of DFU across racial/ethnic cohorts showed increased risk of TW-DED, suggesting that disparities in the risk of DFU and DN appeared to underlie the racial differences we observed in TW-DED progression.

Over the past decade, DR incidence and progression have increased disproportionately among racial and ethnic minorities, with a 2-fold increase among Black and Hispanic populations compared to their White counterparts [26, 27]. Consistent with prior database studies, our results show that Hispanic individuals with NPDR were 40% more likely to progress to TW-DED, while Black individuals had a 15% increased risk compared to the White cohort [9, 28–30]. Independent studies of the Indian Health Service have found increased DR incidence and progression among American Indian and Alaskan Native (AIAN) populations, aligning with the 13% increased TW-DED risk observed in our Other cohort [31, 32]. While reduced access to ophthalmologic services and lower adherence to guideline-recommended eye exams have been implicated in these disparities, our study underscores that inequities persist even in the context of higher treatment utilisation among non-White patients [8, 33]. Of note, a 2025 meta-analysis found no significant difference in the overall prevalence of DR, PDR, or DME between AIAN and non-AIAN groups, although the included studies were limited by heterogeneity and low-quality evidence [34]. This suggests that while some datasets, including ours, indicate elevated risk among such populations, variability in study design and access to care may contribute to inconsistent findings. Overall, these results highlight the need to address structural and access-related factors that likely underlie persistent inequities in DED. Interestingly, Asian individuals exhibited similar TW-DED progression rates to White individuals, though subgroup-specific differences persist. For instance, South Asians have been found to have an increased risk of DR, whereas Chinese Americans exhibit lower DR prevalence, highlighting the need for more granular racial and ethnic categorisation in future research [9, 35, 36].

A key finding of our study is that differences in the burden of DN and DFU may partly explain the observed racial and ethnic differences in TW-DED. Microvascular comorbidities were highly prevalent at baseline, with 35% of patients having DN and 13% having DFU, and both conditions markedly increased TW-DED risk even after adjusting for race and ethnicity. Among White, Hispanic, Black, and Other patients, the presence of DFU conferred over 20% additive hazard on TW-DED progression in those with DN, suggesting a synergistic interaction between these microvascular complications. In White, Hispanic, and Black patients with DFU, DN contributed over 10% additive hazard on TW-DED progression. Our findings suggest that differential distribution and combined effects of microvascular complications may contribute to racial and ethnic differences in the progression of diabetic eye disease. While prior studies have described racial disparities in TW-DED progression, our work highlights DFU and DN as markers of systemic microvascular disease that may help explain these differences [37, 38]. Racial and ethnic differences in the prevalence, severity, and management of DFU and DN are well established. Hispanic, Black, and Native American populations experience disproportionately worse DFU outcomes, including higher rates of ulcer-related morbidity and amputation [39, 40]. Meanwhile, DN may also progress more aggressively in Black and Hispanic populations, often leading to higher incidence of end-stage renal disease [12, 21, 41]. These patterns suggest that racial and ethnic differences in the prevalence, severity, and management of DN and DFU may further accelerate DR progression and meaningfully influence ocular outcomes in a population already burdened by heightened microvascular risk. Ophthalmologists should consider incorporating DFU and DN into DR risk assessment and recognise that improving access to foot and kidney care may have important downstream benefits for preventing vision-threatening disease.

Several limitations of our study must be acknowledged. As an observational study, causal inference cannot be established, and variations in diagnostic and treatment practices across healthcare settings may limit generalisability. The use of coded EHR data introduces potential misclassification bias, as race and ethnicity were classified based on how they were recorded within the EHR. Indicators of social determinants of health, such as insurance status and socioeconomic status, were unavailable, limiting assessment of the impact of structural contributors. DFU and DN were defined using single diagnostic codes (e.g., DN defined by E11.2) rather than composite codes due to the pre-specified analytic structure within the TriNetX platform; this approach may therefore underestimate the true prevalence or severity of these conditions. Additionally, granular data such as diabetes duration or clinical severity of DFU and DN were unavailable, limiting stratification by disease severity, which may influence DR progression. Although GLP-1 RAs have been reported in some studies to accelerate DR progression, our cohorts were well balanced for GLP-1 use, and more recent evidence, including a 2025 systematic review of randomised controlled trials and observational studies and a multidatabase study in the Observational Health Data Sciences and Informatics Evidence Network, found no significant association between GLP-1 therapy and DR risk [42, 43]. Despite well-balanced cohorts after propensity score matching, residual confounding from unmeasured variables remains possible. Future studies may address these limitations by incorporating more granular clinical data and social determinants of health to further explore mechanisms underlying observed differences in progression to TW-DED.

## Conclusions

In this large national study of patients with NPDR, racial and ethnic minority groups experienced a disproportionate burden of progression to TW-DED. DFU and DN were independently associated with increased risk, with foot ulcers showing the strongest association. Adjustment for these microvascular complications attenuated racial and ethnic differences in TW-DED progression, suggesting that variation in the prevalence and management of microvascular comorbidities may contribute to observed differences in DR outcomes. Future efforts should prioritise targeted, intensified multidisciplinary screening and intervention for NPDR patients with DFU and DN to effectively reduce diabetes-related visual morbidity and advance equity in diabetic eye care.

## Summary

### What was known before:

- Racial differences exist in the risk of diabetes-related microvascular complications, including diabetic retinopathy (DR), nephropathy (DN), and foot ulcers (DFU).
- DN and DFU are associated with DR, but their role in its progression to treatment-warranted diabetic eye disease (TW-DED), particularly across racial and ethnic groups, remained unclear.

### What this study adds:

- Hispanic, Black, and Other groups had higher risk of progression to TW-DED, but differences diminished after comorbidity adjustment.
- Racial disparities attenuated after adjusting for DFU and DN burden.
- DFU and DN independently predicted TW-DED progression, with DFU posing greater risk across racial groups. Interacting comorbid microvascular disease burden and differences in management may help explain racial differences in DR outcomes.

## Supplementary information

Supplemental Tables 1-6

Supplemental Tables 7-8

## Data Availability

The data used in this study were sourced from the TriNetX, LLC research network and are subject to licensing and third-party access restrictions. Researchers may request access to TriNetX at https://live.trinetx.com, which may involve licensing costs, a formal data use agreement, and compliance with applicable privacy regulations to ensure that no patient-identifiable information is shared.
