# Supplementary material for: Racial and microvascular determinants of progression to treatment-warranted diabetic eye disease
Source: Eye (Lond). 2026 Apr 7;40(9):1371–7. doi: 10.1038/s41433-026-04417-0 (PMC13269473; doi:10.1038/s41433-026-04417-0)
Supplement: Supplementary file 1 — Supplemental Tables 1-6 [file 41433_2026_4417_MOESM1_ESM.docx]

| **Supplemental Table 1.** Demographic, Diagnostic, Procedure, Medication, Laboratory, and Visit Codes Used in the Definition of Covariates | | |
| --- | --- | --- |
| **Category** | **Code** | **Description** |
| Demographics | AI (TriNetX curated) | Age at Index |
| Demographics | 2106-3 (TriNetX curated) | White |
| Demographics | 1002-5 (TriNetX curated) | American Indian or Alaska Native |
| Demographics | F (TriNetX curated) | Female |
| Demographics | 2076-8 (TriNetX curated) | Native Hawaiian or Other Pacific Islander |
| Demographics | 2135-2 (TriNetX curated) | Hispanic or Latino |
| Demographics | 2054-5 (TriNetX curated) | Black or African-American |
| Demographics | 2186-5 (TriNetX curated) | Not Hispanic or Latino |
| Demographics | 2131-1 (TriNetX curated) | Other Race |
| Demographics | 2028-9 (TriNetX curated) | Asian |
| Diagnosis (ICD-10) | E11.32 | Type 2 diabetes mellitus with mild nonproliferative diabetic retinopathy |
| Diagnosis (ICD-10) | E11.33 | Type 2 diabetes mellitus with moderate nonproliferative diabetic retinopathy |
| Diagnosis (ICD-10) | E11.34 | Type 2 diabetes mellitus with severe nonproliferative diabetic retinopathy |
| Diagnosis (ICD-10) | E11.35 | Type 2 diabetes mellitus with proliferative diabetic retinopathy |
| Diagnosis (ICD-10) | E11.311, E11.321, E11.331, E11.341, E11.351 | Type 2 diabetes mellitus with macular oedema |
| Diagnosis (ICD-10) | H43.13, H43.399 | Vitreous haemorrhage |
| Diagnosis (ICD-10) | H54, H44.52 | Blindness or low vision |
| Diagnosis (ICD-10) | E11.51 | Type 2 diabetes mellitus with diabetic peripheral angiopathy without gangrene |
| Diagnosis (ICD-10) | E11.52 | Type 2 diabetes mellitus with diabetic peripheral angiopathy with gangrene |
| Diagnosis (ICD-10) | L97 | Non-pressure chronic ulcer of lower limb (DFU) |
| Diagnosis (ICD-10) | E11.2 | Type 2 diabetes mellitus with kidney complications (DN) |
| Diagnosis (ICD-10) | N18.6 | End stage renal disease |
| Diagnosis (ICD-10) | E11.42 | Type 2 diabetes mellitus with diabetic polyneuropathy |
| Diagnosis (ICD-10) | I10-I1A | Hypertensive diseases |
| Diagnosis (ICD-10) | J40-J4A | Chronic lower respiratory diseases |
| Diagnosis (ICD-10) | I20-I25 | Ischemic heart diseases |
| Diagnosis (ICD-10) | I50 | Heart failure |
| Diagnosis (ICD-10) | I48 | Atrial fibrillation and flutter |
| Diagnosis (ICD-10) | I60-I69 | Cerebrovascular diseases |
| Diagnosis (ICD-10) | I74 | Arterial embolism and thrombosis |
| Diagnosis (ICD-10) | I70 | Atherosclerosis |
| Diagnosis (ICD-10) | I73 | Other peripheral vascular diseases |
| Diagnosis (ICD-10) | I77 | Other disorders of arteries and arterioles |
| Diagnosis (ICD-10) | C00-D49 | Neoplasms |
| Diagnosis (ICD-10) | Z55-Z65 | Potential health hazards related to socioeconomic and psychosocial circumstances |
| Diagnosis (ICD-10) | K74 | Fibrosis and cirrhosis of liver |
| Diagnosis (ICD-10) | K74.6 | Other and unspecified cirrhosis of liver |
| Diagnosis (ICD-10) | N18 | Chronic kidney disease (CKD) |
| Diagnosis (ICD-10) | Z72.0 | Tobacco use |
| Procedure (CPT) | 67028 | Intravitreal injection |
| Procedure (CPT) | 67228, 67210 | Panretinal or focal photocoagulation |
| Procedure (CPT) | 67036, 67039, 67040, 67041, 67042, 67043, 67108, 67113 | Pars plana vitrectomy |
| Medication (ATC) | C03 | Diuretics |
| Medication (ATC) | C07 | Beta blocking agents |
| Medication (ATC) | C09A | Ace inhibitors |
| Medication (ATC) | C09C | Angiotensin II receptor blockers |
| Medication (ATC) | C08 | Calcium channel blockers |
| Medication (ATC) | C10 | Lipid modifying agents |
| Medication (ATC) | B01 | Antithrombotic agents |
| Medication (ATC) | A10A | Insulins and analogues |
| Medication (ATC) | A10B | Other blood glucose lowering drugs (including metformin) |
| Laboratory | 9037 (TriNetX curated) | Haemoglobin A1c |
| Laboratory | 9083 (TriNetX curated) | Body mass index |
| Laboratory | 8001 (TriNetX curated) | Glomerular filtration rate |
| Laboratory | 9000 (TriNetX curated) | Cholesterol [Mass/volume] in Serum or Plasma |
| Laboratory | 9004 (TriNetX curated) | Triglyceride [Mass/volume] in Serum, Plasma or Blood |
| Visit | AMB (TriNetX curated) | Visit: Ambulatory |
| Visit | IMP (TriNetX curated) | Visit: Inpatient Encounter |
| Visit | EMER (TriNetX curated) | Visit: Emergency |
| ATC, Anatomical Therapeutic Chemical Classification; CPT, Current Procedural Terminology; ICD-10, International Statistical Classification of Diseases and Related Health Problems, Tenth Revision; DFU, diabetic foot ulcer; DN, diabetic nephropathy. | | |

| **Supplemental Table 2.** Baseline Characteristics of Non-Hispanic White and Hispanic Individuals with Non-Proliferative Diabetic Retinopathy Before and After Propensity Score Matching | | | | | | | |
| --- | --- | --- | --- | --- | --- | --- | --- |
|  | **Before propensity score matching, No. (%)** | | |  | **After propensity score matching, No. (%)** | | |
| **Characteristic** | **Hispanic**  **(N = 19,624)** | **White**  **(N = 62,101)** | **SMD** |  | **Hispanic (N = 16,916)** | **White (N = 16,916)** | **SMD** |
| **Demographics** |  |  |  |  |  |  |  |
| Age at index, years, mean ± SD | 56.7 ± 11.9 | 63.8 ± 12.5 | 0.603 |  | 58.1 ± 11.5 | 57.5 ± 13.6 | 0.050 |
| Female | 10,467 (53.4) | 28,349 (45.7) | 0.149 |  | 8,688 (51.4) | 8,724 (51.6) | 0.004 |
| **Comorbidities** |  |  |  |  |  |  |  |
| Type 2 diabetes mellitus with diabetic peripheral angiopathy without gangrene | 1,015 (5.2) | 3,687 (5.9) | 0.051 |  | 855 (5.1) | 837 (4.9) | 0.005 |
| Type 2 diabetes mellitus with diabetic peripheral angiopathy with gangrene | 198 (1.0) | 520 (0.8) | 0.014 |  | 166 (1.0) | 161 (1.0) | 0.003 |
| Type 2 diabetes mellitus with diabetic polyneuropathy | 3,182 (16.2) | 11,529 (18.6) | 0.086 |  | 2,739 (16.2) | 2,803 (16.6) | 0.010 |
| Non-pressure chronic ulcer of lower limb (DFU) | 1,331 (6.8) | 4,760 (7.7) | 0.043 |  | 1,151 (6.8) | 1,143 (6.8) | 0.002 |
| End stage renal disease | 673 (3.4) | 1,605 (2.6) | 0.048 |  | 567 (3.4) | 555 (3.3) | 0.004 |
| Chronic kidney disease | 2,692 (13.7) | 12,923 (20.8) | 0.200 |  | 2,447 (14.5) | 2,496 (14.8) | 0.008 |
| Type 2 diabetes mellitus with diabetic kidney complications (DN) | 1,696 (8.6) | 4,819 (7.8) | 0.025 |  | 1,356 (8.0) | 1,358 (8.0) | <0.001 |
| Hypertensive diseases | 12,838 (65.4) | 43,126 (69.5) | 0.124 |  | 10,948 (64.7) | 11,135 (65.8) | 0.023 |
| Chronic lower respiratory diseases | 2,587 (13.2) | 14,160 (22.8) | 0.277 |  | 2,460 (14.5) | 2,661 (15.7) | 0.033 |
| Ischemic heart diseases | 3,204 (16.3) | 17,576 (28.3) | 0.307 |  | 2,978 (17.6) | 3,084 (18.2) | 0.016 |
| Heart failure | 1,735 (8.8) | 8,931 (14.4) | 0.189 |  | 1,580 (9.3) | 1,659 (9.8) | 0.016 |
| Atrial fibrillation and flutter | 697 (3.6) | 7,098 (11.4) | 0.314 |  | 690 (4.1) | 733 (4.3) | 0.013 |
| Cerebrovascular diseases | 1,860 (9.5) | 8,909 (14.4) | 0.156 |  | 1,677 (9.9) | 1,777 (10.5) | 0.020 |
| Arterial embolism and thrombosis | 149 (0.8) | 686 (1.1) | 0.041 |  | 135 (0.8) | 147 (0.9) | 0.008 |
| Atherosclerosis | 1,305 (6.7) | 5,443 (8.8) | 0.098 |  | 1,162 (6.9) | 1,148 (6.8) | 0.003 |
| Other peripheral vascular diseases | 1,442 (7.4) | 6,330 (10.2) | 0.116 |  | 1,266 (7.5) | 1,285 (7.6) | 0.004 |
| Other disorders of arteries and arterioles | 576 (2.9) | 3,186 (5.1) | 0.119 |  | 521 (3.1) | 550 (3.3) | 0.010 |
| Neoplasms | 4,999 (25.5) | 21,504 (34.7) | 0.239 |  | 4,523 (26.7) | 4,550 (26.9) | 0.004 |
| Potential health hazards related to socioeconomic and psychosocial circumstances | 1,186 (6.0) | 2,303 (3.7) | 0.084 |  | 938 (5.5) | 979 (5.8) | 0.010 |
| Tobacco use | 875 (4.5) | 3,176 (5.1) | 0.041 |  | 797 (4.7) | 860 (5.1) | 0.017 |
| Fibrosis and cirrhosis of liver | 749 (3.8) | 1,790 (2.9) | 0.047 |  | 605 (3.6) | 626 (3.7) | 0.007 |
| Other and unspecified cirrhosis of liver | 704 (3.8) | 1,608 (2.6) | 0.047 |  | 566 (3.3) | 575 (3.4) | 0.003 |
| **Medications** |  |  |  |  |  |  |  |
| Insulins and analogues | 7,548 (38.5) | 31,274 (50.4) | 0.212 |  | 6,616 (39.1) | 6,775 (40.1) | 0.019 |
| Blood glucose lowering drugs (including metformin) | 10,572 (53.9) | 36,047 (58.1) | 0.061 |  | 8,914 (52.7) | 9,000 (53.2) | 0.010 |
| Diuretics | 5,450 (27.8) | 24,760 (39.9) | 0.253 |  | 4,873 (28.8) | 4,868 (28.8) | 0.001 |
| Beta blocking agents | 4,700 (24.0) | 25,966 (41.8) | 0.384 |  | 4,400 (26.0) | 4,467 (26.4) | 0.009 |
| ACE inhibitors | 7,044 (35.9) | 25,411 (41.0) | 0.092 |  | 6,022 (35.6) | 6,122 (36.2) | 0.012 |
| Angiotensin II receptor blockers | 2,710 (13.8) | 14,469 (23.3) | 0.238 |  | 2,533 (15.0) | 2,597 (15.4) | 0.011 |
| Calcium channel blockers | 3,500 (17.8) | 17,760 (28.6) | 0.255 |  | 3,230 (19.1) | 3,284 (19.4) | 0.008 |
| Lipid modifying agents | 9,316 (47.5) | 37,454 (60.4) | 0.234 |  | 8,179 (48.4) | 8,244 (48.7) | 0.008 |
| Antithrombotic agents | 6,157 (31.4) | 29,717 (47.9) | 0.325 |  | 5,561 (32.9) | 5,699 (33.7) | 0.017 |
| **Laboratory values, mean ± SD** |  |  |  |  |  |  |  |
| Haemoglobin A1c (%) | 8.7 ± 2.2 | 7.8 ± 1.9 | 0.431 |  | 8.5 ± 2.1 | 8.3 ± 2.2 | 0.117 |
| BMI (kg/m^2^) | 31.3 ± 6.8 | 33.0 ± 7.4 | 0.242 |  | 31.4 ± 6.8 | 32.4 ± 8.0 | 0.124 |
| Glomerular filtration rate (mL/min/1.73 m²) | 79.0 ± 34.5 | 71.5 ± 28.2 | 0.244 |  | 77.6 ± 33.5 | 79.5 ± 31.0 | 0.059 |
| Total cholesterol (mg/dL) | 167.9 ± 49.5 | 160.1 ± 47.6 | 0.155 |  | 166.2 ± 48.5 | 168.7 ± 53.4 | 0.049 |
| Total triglycerides (mg/dL) | 179.4 ± 161.5 | 172.3 ± 160.2 | 0.040 |  | 178.3 ± 160.4 | 186.5 ± 221.8 | 0.042 |
| **Healthcare utilisation** |  |  |  |  |  |  |  |
| Ambulatory Visit | 14,228 (72.5) | 50,261 (81.0) | 0.127 |  | 11,795 (69.7) | 11,848 (70.0) | 0.007 |
| Emergency Department Visit | 6,524 (33.3) | 23,684 (38.2) | 0.036 |  | 5,329 (31.5) | 5,442 (32.2) | 0.014 |
| Inpatient Visit | 6,402 (32.6) | 24,485 (39.5) | 0.180 |  | 4,751 (28.1) | 4,718 (27.9) | 0.004 |
| SMD, standardised mean difference; SD, standard deviation; BMI, body mass index; DFU, diabetic foot ulcer; DN, diabetic nephropathy. | | | | | | | |

| **Supplemental Table 3.** Baseline Characteristics of White and Black Individuals with Non-Proliferative Diabetic Retinopathy Before and After Propensity Score Matching | | | | | | | |
| --- | --- | --- | --- | --- | --- | --- | --- |
|  | **Before propensity score matching, No. (%)** | | |  | **After propensity score matching, No. (%)** | | |
| **Characteristic** | **Black**  **(N = 32,376)** | **White**  **(N = 62,101)** | **SMD** |  | **Black**  **(N =** **28,441)** | **White**  **(N = 28,441)** | **SMD** |
| **Demographics** |  |  |  |  |  |  |  |
| Age at index, years, mean ± SD | 60.0 ± 12.4 | 63.8 ± 12.5 | 0.301 |  | 60.9 ± 12.1 | 60.7 ± 13.4 | 0.011 |
| Female | 18,856 (58.3) | 28,349 (45.7) | 0.254 |  | 15,871 (55.8) | 15,967 (56.1) | 0.007 |
| **Comorbidities** |  |  |  |  |  |  |  |
| Type 2 diabetes mellitus with diabetic peripheral angiopathy without gangrene | 1,759 (5.4) | 3,687 (5.9) | 0.022 |  | 1,549 (5.4) | 1,539 (5.4) | 0.002 |
| Type 2 diabetes mellitus with diabetic peripheral angiopathy with gangrene | 334 (1.0) | 520 (0.8) | 0.020 |  | 276 (1.0) | 266 (0.9) | 0.004 |
| Type 2 diabetes mellitus with diabetic polyneuropathy | 5,276 (16.3) | 11,529 (18.6) | 0.060 |  | 4,658 (16.4) | 4,582 (16.1) | 0.007 |
| Non-pressure chronic ulcer of lower limb (DFU) | 1,848 (5.7) | 4,760 (7.7) | 0.079 |  | 1,677 (5.9) | 1,621 (5.7) | 0.008 |
| End stage renal disease | 1,720 (5.3) | 1,605 (2.6) | 0.140 |  | 1,183 (4.2) | 1,168 (4.1) | 0.003 |
| Chronic kidney disease | 7,706 (23.8) | 12,923 (20.8) | 0.072 |  | 6,325 (22.2) | 6,220 (21.9) | 0.009 |
| Type 2 diabetes mellitus with diabetic kidney complications (DN) | 2,653 (8.2) | 4,819 (7.8) | 0.016 |  | 2,211 (7.8) | 2,184 (7.7) | 0.004 |
| Hypertensive diseases | 24,214 (74.8) | 43,126 (69.5) | 0.119 |  | 20,616 (72.5) | 20,297 (71.4) | 0.025 |
| Chronic lower respiratory diseases | 6,953 (21.5) | 14,160 (22.8) | 0.032 |  | 6,075 (21.4) | 6,054 (21.3) | 0.002 |
| Ischemic heart diseases | 6,625 (20.5) | 17,576 (28.3) | 0.184 |  | 6,105 (21.5) | 6,088 (21.4) | 0.001 |
| Heart failure | 5,127 (15.8) | 8,931 (14.4) | 0.041 |  | 4,250 (14.9) | 4,158 (14.6) | 0.009 |
| Atrial fibrillation and flutter | 1,994 (6.2) | 7,098 (11.4) | 0.187 |  | 1,914 (6.7) | 1,879 (6.6) | 0.005 |
| Cerebrovascular diseases | 4,472 (13.8) | 8,909 (14.4) | 0.015 |  | 3,863 (13.6) | 3,781 (13.3) | 0.008 |
| Arterial embolism and thrombosis | 359 (1.1) | 686 (1.1) | <0.001 |  | 305 (1.1) | 299 (1.1) | 0.002 |
| Atherosclerosis | 2,612 (8.1) | 5,443 (8.8) | 0.025 |  | 2,298 (8.1) | 2,253 (7.9) | 0.006 |
| Other peripheral vascular diseases | 3,004 (9.3) | 6,330 (10.2) | 0.031 |  | 2,641 (9.3) | 2,588 (9.1) | 0.006 |
| Other disorders of arteries and arterioles | 1,446 (4.5) | 3,186 (5.1) | 0.031 |  | 1,266 (4.5) | 1,268 (4.5) | <0.001 |
| Neoplasms | 9,398 (29.0) | 21,504 (34.7) | 0.121 |  | 8,405 (29.6) | 8,252 (29.0) | 0.012 |
| Persons with potential health hazards related to socioeconomic and psychosocial circumstances | 1,498 (4.6) | 2,303 (3.7) | 0.046 |  | 1,234 (4.3) | 1,246 (4.4) | 0.002 |
| Tobacco use | 1,984 (6.1) | 3,176 (5.1) | 0.044 |  | 1,652 (5.8) | 1,678 (5.9) | 0.004 |
| Fibrosis and cirrhosis of liver | 615 (1.9) | 1,790 (2.9) | 0.064 |  | 578 (2.0) | 575 (2.0) | 0.001 |
| Other and unspecified cirrhosis of liver | 539 (1.7) | 1,608 (2.6) | 0.064 |  | 512 (1.8) | 505 (1.8) | 0.002 |
| **Medications** |  |  |  |  |  |  |  |
| Insulins and analogues | 17,252 (53.3) | 31,274 (50.4) | 0.058 |  | 14,502 (51.0) | 14,232 (50.0) | 0.019 |
| Blood glucose lowering drugs (including metformin) | 19,496 (60.2) | 36,047 (58.1) | 0.044 |  | 16,573 (58.3) | 16,321 (57.4) | 0.018 |
| Diuretics | 15,641 (48.3) | 24,760 (39.9) | 0.170 |  | 12,830 (45.1) | 12,551 (44.1) | 0.020 |
| Beta blocking agents | 13,964 (43.2) | 25,966 (41.8) | 0.026 |  | 11,757 (41.3) | 11,519 (40.5) | 0.017 |
| ACE inhibitors | 14,137 (43.7) | 25,411 (41.0) | 0.055 |  | 11,879 (41.8) | 11,588 (40.7) | 0.021 |
| Angiotensin II receptor blockers | 8,950 (27.7) | 14,469 (23.3) | 0.100 |  | 7,354 (25.9) | 7,264 (25.5) | 0.007 |
| Calcium channel blockers | 13,673 (42.3) | 17,760 (28.6) | 0.288 |  | 10,715 (37.7) | 10,421 (36.6) | 0.021 |
| Lipid modifying agents | 19,234 (59.4) | 37,454 (60.4) | 0.019 |  | 16,581 (58.3) | 16,241 (57.1) | 0.024 |
| Antithrombotic agents | 16,208 (50.1) | 29,717 (47.9) | 0.044 |  | 13,676 (48.1) | 13,468 (47.4) | 0.015 |
| **Laboratory values, mean ± SD** |  |  |  |  |  |  |  |
| Haemoglobin A1c (%) | 8.4 ± 2.5 | 7.8 ± 1.9 | 0.247 |  | 8.2 ± 2.4 | 8.1 ± 2.1 | 0.076 |
| BMI (kg/m²) | 33.3 ± 8.0 | 33.0 ± 7.4 | 0.037 |  | 33.2 ± 7.8 | 33.3 ± 7.9 | 0.021 |
| Glomerular filtration rate (mL/min/1.73 m²) | 75.3 ± 35.6 | 71.5 ± 28.2 | 0.121 |  | 75.1 ± 34.3 | 73.5 ± 31.4 | 0.050 |
| Total cholesterol (mg/dL) | 167.9 ± 50.8 | 160.1 ± 47.6 | 0.158 |  | 166.7 ± 50.1 | 166.2 ± 50.8 | 0.010 |
| Total triglycerides (mg/dL) | 131.9 ± 111.1 | 172.3 ± 160.2 | 0.293 |  | 129.9 ± 110.4 | 133.2 ± 110.6 | 0.042 |
| **Healthcare Utilisation** |  |  |  |  |  |  |  |
| Ambulatory Visit | 26,117 (80.7) | 50,261 (81.0) | 0.007 |  | 22,681 (79.7) | 22,442 (78.9) | 0.021 |
| Emergency Department Visit | 15,412 (47.6) | 23,684 (38.2) | 0.192 |  | 12,631 (44.4) | 12,446 (43.8) | 0.013 |
| Inpatient Visit | 15,396 (47.6) | 24,485 (39.5) | 0.164 |  | 12,671 (44.6) | 12,561 (44.2) | 0.008 |
| SMD, standardised mean difference; SD, standard deviation; BMI, body mass index; DFU, diabetic foot ulcer; DN, diabetic nephropathy. | | | | | | | |

| **Supplemental Table 4.** Baseline Characteristics of Asian and White Individuals with Non-Proliferative Diabetic Retinopathy Before and After Propensity Score Matching | | | | | | | |
| --- | --- | --- | --- | --- | --- | --- | --- |
|  | **Before propensity score matching, No. (%)** | | |  | **After propensity score matching, No. (%)** | | |
| **Characteristic** | **Asian**  **(N = 8,357)** | **White**  **(N = 62,101)** | **SMD** |  | **Asian**  **(N = 8,248)** | **White**  **(N = 8,248)** | **SMD** |
| **Demographics** |  |  |  |  |  |  |  |
| Age at index, years, mean ± SD | 62.7 ± 12.2 | 63.8 ± 12.5 | 0.084 |  | 62.7 ± 12.2 | 62.4 ± 13.4 | 0.023 |
| Female | 3,882 (46.5) | 28,349 (45.7) | 0.016 |  | 3,825 (46.4) | 3,779 (45.8) | 0.011 |
| **Comorbidities** |  |  |  |  |  |  |  |
| Type 2 diabetes mellitus with diabetic peripheral angiopathy without gangrene | 207 (2.5) | 3,687 (5.9) | 0.173 |  | 206 (2.5) | 230 (2.8) | 0.018 |
| Type 2 diabetes mellitus with diabetic peripheral angiopathy with gangrene | 28 (0.3) | 520 (0.8) | 0.066 |  | 27 (0.3) | 31 (0.4) | 0.008 |
| Type 2 diabetes mellitus with diabetic polyneuropathy | 686 (8.2) | 11,529 (18.6) | 0.308 |  | 684 (8.3) | 655 (7.9) | 0.013 |
| Non-pressure chronic ulcer of lower limb (DFU) | 225 (2.7) | 4,760 (7.7) | 0.226 |  | 224 (2.7) | 240 (2.9) | 0.012 |
| Chronic kidney disease | 1,751 (21.0) | 12,923 (20.8) | 0.004 |  | 1,693 (20.5) | 1,663 (20.2) | 0.009 |
| End stage renal disease | 416 (5.0) | 1,605 (2.6) | 0.126 |  | 380 (4.6) | 397 (4.8) | 0.010 |
| Type 2 diabetes mellitus with diabetic kidney complications (DN) | 677 (8.1) | 4,819 (7.8) | 0.013 |  | 648 (7.9) | 617 (7.5) | 0.014 |
| Hypertensive diseases | 5,527 (66.2) | 43,126 (69.5) | 0.071 |  | 5,432 (65.9) | 5,239 (63.5) | 0.049 |
| Chronic lower respiratory diseases | 1,303 (15.6) | 14,160 (22.8) | 0.184 |  | 1,294 (15.7) | 1,312 (15.9) | 0.006 |
| Ischemic heart diseases | 1,725 (20.7) | 17,576 (28.3) | 0.179 |  | 1,699 (20.6) | 1,626 (19.7) | 0.022 |
| Heart failure | 721 (8.6) | 8,931 (14.4) | 0.181 |  | 706 (8.6) | 699 (8.5) | 0.003 |
| Atrial fibrillation and flutter | 450 (5.4) | 7,098 (11.4) | 0.219 |  | 445 (5.4) | 453 (5.5) | 0.004 |
| Cerebrovascular diseases | 1,009 (12.1) | 8,909 (14.4) | 0.067 |  | 991 (12.0) | 978 (11.9) | 0.005 |
| Arterial embolism and thrombosis | 47 (0.6) | 686 (1.1) | 0.060 |  | 46 (0.6) | 43 (0.5) | 0.005 |
| Atherosclerosis | 581 (7.0) | 5,443 (8.8) | 0.067 |  | 570 (6.9) | 561 (6.8) | 0.004 |
| Other peripheral vascular diseases | 394 (4.7) | 6,330 (10.2) | 0.210 |  | 393 (4.8) | 401 (4.9) | 0.005 |
| Other disorders of arteries and arterioles | 304 (3.6) | 3,186 (5.1) | 0.073 |  | 296 (3.6) | 291 (3.5) | 0.003 |
| Neoplasms | 2,215 (26.5) | 21,504 (34.7) | 0.177 |  | 2,194 (26.6) | 2,082 (25.2) | 0.031 |
| Potential health hazards related to socioeconomic and psychosocial circumstances | 200 (2.4) | 2,303 (3.7) | 0.077 |  | 199 (2.4) | 187 (2.3) | 0.010 |
| Tobacco use | 217 (2.6) | 3,176 (5.1) | 0.131 |  | 217 (2.6) | 208 (2.5) | 0.007 |
| Fibrosis and cirrhosis of liver | 179 (2.1) | 1,790 (2.9) | 0.047 |  | 173 (2.1) | 161 (2.0) | 0.010 |
| Other and unspecified cirrhosis of liver | 155 (1.9) | 1,608 (2.6) | 0.050 |  | 150 (1.8) | 138 (1.7) | 0.011 |
| **Medications** |  |  |  |  |  |  |  |
| Insulins and analogues | 3,340 (40.0) | 31,274 (50.4) | 0.210 |  | 3,292 (39.9) | 3,212 (38.9) | 0.020 |
| Blood glucose lowering drugs (including metformin) | 5,050 (60.5) | 36,047 (58.1) | 0.049 |  | 4,959 (60.1) | 4,743 (57.5) | 0.053 |
| Diuretics | 2,449 (29.3) | 24,760 (39.9) | 0.224 |  | 2,414 (29.3) | 2,342 (28.4) | 0.019 |
| Beta blocking agents | 2,848 (34.1) | 25,966 (41.8) | 0.160 |  | 2,796 (33.9) | 2,693 (32.7) | 0.027 |
| ACE inhibitors | 2,555 (30.6) | 25,411 (41.0) | 0.217 |  | 2,535 (30.7) | 2,407 (29.2) | 0.034 |
| Angiotensin II receptor blockers | 2,586 (31.0) | 14,469 (23.3) | 0.173 |  | 2,507 (30.4) | 2,442 (29.6) | 0.017 |
| Calcium channel blockers | 2,391 (28.6) | 17,760 (28.6) | <0.001 |  | 2,322 (28.2) | 2,222 (26.9) | 0.027 |
| Lipid modifying agents | 4,808 (57.6) | 37,454 (60.4) | 0.056 |  | 4,729 (57.3) | 4,526 (54.9) | 0.050 |
| Antithrombotic agents | 3,227 (38.7) | 29,717 (47.9) | 0.187 |  | 3,178 (38.5) | 3,047 (36.9) | 0.033 |
| **Laboratory values, mean ± SD** |  |  |  |  |  |  |  |
| Haemoglobin A1c (%) | 7.9 ± 1.8 | 7.8 ± 1.9 | 0.059 |  | 7.9 ± 1.8 | 7.9 ± 1.9 | 0.033 |
| BMI (kg/m²) | 27.4 ± 5.4 | 33.0 ± 7.4 | 0.866 |  | 27.4 ± 5.4 | 28.6 ± 6.7 | 0.195 |
| Glomerular filtration rate (mL/min/1.73 m²) | 75.0 ± 33.9 | 71.5 ± 28.2 | 0.112 |  | 75.3 ± 33.7 | 75.0 ± 32.0 | 0.008 |
| Cholesterol (mg/dL) | 163.2 ± 48.1 | 160.1 ± 47.6 | 0.064 |  | 163.1 ± 47.9 | 163.9 ± 46.2 | 0.016 |
| Triglycerides (mg/dL) | 169.1 ± 137.4 | 172.3 ± 160.2 | 0.022 |  | 169.1 ± 138.0 | 165.3 ± 142.1 | 0.027 |
| **Healthcare Utilisation** |  |  |  |  |  |  |  |
| Ambulatory Visit | 7,029 (84.2) | 50,261 (81.0) | 0.084 |  | 6,928 (84.0) | 6,829 (82.8) | 0.032 |
| Emergency Department Visit | 3,218 (38.5) | 23,684 (38.2) | 0.008 |  | 3,154 (38.2) | 3,090 (37.5) | 0.016 |
| Inpatient Visit | 3,007 (36.0) | 24,485 (39.5) | 0.071 |  | 2,951 (35.8) | 2,889 (35.0) | 0.016 |
| SMD, standardised mean difference; SD, standard deviation; BMI, body mass index; DFU, diabetic foot ulcer; DN, diabetic nephropathy. | | | | | | | |

| **Supplemental Table 5.** Baseline Characteristics of Non-Hispanic White and Non-Hispanic Other Individuals with Non-Proliferative Diabetic Retinopathy Before and After Propensity Score Matching | | | | | | | |
| --- | --- | --- | --- | --- | --- | --- | --- |
|  | **Before propensity score matching, No. (%)** | | |  | **After propensity score matching, No. (%)** | | |
| **Characteristic** | **Other**  **(N = 7,544)** | **White**  **(N = 62,101)** | **SMD** |  | **Other**  **(N = 7,174)** | **White**  **(N = 7,174)** | **SMD** |
| **Demographics** |  |  |  |  |  |  |  |
| Age at index, years, mean ± SD | 62.2 ± 12.4 | 63.8 ± 12.5 | 0.141 |  | 61.9 ± 12.5 | 61.8 ± 13.2 | 0.005 |
| Female | 3,072 (40.8) | 28,349 (45.7) | 0.095 |  | 3,003 (41.9) | 3,028 (42.2) | 0.007 |
| **Comorbidities** |  |  |  |  |  |  |  |
| Type 2 diabetes mellitus with diabetic peripheral angiopathy without gangrene | 554 (7.4) | 3,687 (5.9) | 0.058 |  | 422 (5.9) | 445 (6.2) | 0.013 |
| Type 2 diabetes mellitus with diabetic peripheral angiopathy with gangrene | 75 (1.0) | 520 (0.8) | 0.019 |  | 55 (0.8) | 54 (0.8) | 0.002 |
| Type 2 diabetes mellitus with diabetic polyneuropathy | 1,342 (17.8) | 11,529 (18.6) | 0.014 |  | 1,169 (16.3) | 1,205 (16.8) | 0.014 |
| Non-pressure chronic ulcer of lower limb (DFU) | 626 (8.3) | 4,760 (7.7) | 0.025 |  | 525 (7.3) | 561 (7.8) | 0.019 |
| Chronic kidney disease | 1,812 (24.0) | 12,923 (20.8) | 0.078 |  | 1,522 (21.2) | 1,712 (23.9) | 0.063 |
| End stage renal disease | 391 (5.2) | 1,605 (2.6) | 0.139 |  | 287 (4.0) | 362 (5.0) | 0.050 |
| Type 2 diabetes mellitus with diabetic kidney complications (DN) | 680 (9.0) | 4,819 (7.8) | 0.040 |  | 535 (7.5) | 617 (8.6) | 0.042 |
| Hypertensive diseases | 5,265 (69.9) | 43,126 (69.5) | 0.014 |  | 4,904 (68.4) | 4,943 (68.9) | 0.012 |
| Chronic lower respiratory diseases | 1,593 (21.1) | 14,160 (22.8) | 0.030 |  | 1,406 (19.6) | 1,478 (20.6) | 0.025 |
| Ischemic heart diseases | 2,130 (28.3) | 17,576 (28.3) | 0.010 |  | 1,842 (25.7) | 1,932 (26.9) | 0.028 |
| Heart failure | 1,143 (15.2) | 8,931 (14.4) | 0.025 |  | 920 (12.8) | 1,024 (14.3) | 0.042 |
| Atrial fibrillation and flutter | 819 (10.9) | 7,098 (11.4) | 0.017 |  | 686 (9.6) | 738 (10.3) | 0.024 |
| Cerebrovascular diseases | 1,090 (14.5) | 8,909 (14.4) | 0.010 |  | 929 (12.9) | 950 (13.2) | 0.009 |
| Arterial embolism and thrombosis | 91 (1.2) | 686 (1.1) | 0.014 |  | 64 (0.9) | 70 (1.0) | 0.009 |
| Atherosclerosis | 705 (9.4) | 5,443 (8.8) | 0.020 |  | 572 (8.0) | 606 (8.4) | 0.017 |
| Other peripheral vascular diseases | 859 (11.4) | 6,330 (10.2) | 0.039 |  | 693 (9.7) | 720 (10.0) | 0.013 |
| Other disorders of arteries and arterioles | 360 (4.8) | 3,186 (5.1) | 0.014 |  | 295 (4.1) | 315 (4.4) | 0.014 |
| Neoplasms | 2,186 (29.0) | 21,504 (34.7) | 0.123 |  | 1,999 (27.9) | 2,019 (28.1) | 0.006 |
| Potential health hazards related to socioeconomic and psychosocial circumstances | 223 (3.0) | 2,303 (3.7) | 0.037 |  | 210 (2.9) | 194 (2.7) | 0.013 |
| Tobacco use | 303 (4.0) | 3,176 (5.1) | 0.043 |  | 278 (3.9) | 260 (3.6) | 0.013 |
| Fibrosis and cirrhosis of liver | 166 (2.2) | 1,790 (2.9) | 0.043 |  | 149 (2.1) | 150 (2.1) | 0.001 |
| Other and unspecified cirrhosis of liver | 151 (2.0) | 1,608 (2.6) | 0.039 |  | 134 (1.9) | 139 (1.9) | 0.005 |
| **Medications** |  |  |  |  |  |  |  |
| Insulins and analogues | 2,534 (33.6) | 31,274 (50.4) | 0.345 |  | 2,530 (35.3) | 2,488 (34.7) | 0.012 |
| Blood glucose lowering drugs (including metformin) | 3,068 (40.7) | 36,047 (58.1) | 0.372 |  | 3,065 (42.7) | 3,014 (42.0) | 0.014 |
| Diuretics | 1,646 (21.8) | 24,760 (39.9) | 0.404 |  | 1,645 (22.9) | 1,738 (24.2) | 0.031 |
| Beta blocking agents | 1,842 (24.4) | 25,966 (41.8) | 0.379 |  | 1,839 (25.6) | 1,886 (26.3) | 0.015 |
| ACE inhibitors | 1,983 (26.3) | 25,411 (41.0) | 0.320 |  | 1,980 (27.6) | 1,962 (27.3) | 0.006 |
| Angiotensin II receptor blockers | 1,238 (16.4) | 14,469 (23.3) | 0.184 |  | 1,235 (17.2) | 1,227 (17.1) | 0.003 |
| Calcium channel blockers | 1,442 (19.1) | 17,760 (28.6) | 0.227 |  | 1,439 (20.1) | 1,428 (19.9) | 0.004 |
| Lipid modifying agents | 2,873 (38.1) | 37,454 (60.4) | 0.473 |  | 2,872 (40.0) | 2,847 (39.7) | 0.007 |
| Antithrombotic agents | 2,308 (30.6) | 29,717 (47.9) | 0.366 |  | 2,303 (32.1) | 2,317 (32.3) | 0.004 |
| **Laboratory values, mean ± SD** |  |  |  |  |  |  |  |
| Haemoglobin A1c (%) | 8.5 ± 2.2 | 7.8 ± 1.9 | 0.338 |  | 8.5 ± 2.2 | 8.2 ± 2.2 | 0.143 |
| BMI (kg/m²) | 31.5 ± 7.3 | 33.0 ± 7.4 | 0.189 |  | 31.6 ± 7.3 | 32.0 ± 7.6 | 0.062 |
| Glomerular filtration rate (mL/min/1.73 m²) | 71.5 ± 33.3 | 71.5 ± 28.2 | 0.008 |  | 71.7 ± 33.1 | 73.1 ± 32.9 | 0.043 |
| Cholesterol (mg/dL) | 167.7 ± 52.0 | 160.1 ± 47.6 | 0.159 |  | 167.7 ± 52.0 | 163.9 ± 48.3 | 0.076 |
| Triglycerides (mg/dL) | 178.3 ± 181.3 | 172.3 ± 160.2 | 0.042 |  | 178.5 ± 181.5 | 175.4 ± 204.5 | 0.016 |
| **Healthcare Utilisation** |  |  |  |  |  |  |  |
| Ambulatory Visit | 6,038 (80.1) | 50,261 (81.0) | 0.002 |  | 5,677 (79.1) | 5,572 (77.7) | 0.036 |
| Emergency Department Visit | 2,280 (30.3) | 23,684 (38.2) | 0.136 |  | 2,262 (31.5) | 2,312 (32.2) | 0.015 |
| Inpatient Visit | 1,972 (26.2) | 24,485 (39.5) | 0.279 |  | 1,961 (27.3) | 1,987 (27.7) | 0.008 |
| SMD, standardised mean difference; SD, standard deviation; BMI, body mass index; DFU, diabetic foot ulcer; DN, diabetic nephropathy. | | | | | | | |

| **Supplemental Table 6.** Multivariable Cox Proportional Hazards Model Identifying Predictors of Treatment-Warranted Diabetic Eye Disease Progression in Patients with Non-Proliferative Diabetic Retinopathy | |
| --- | --- |
| **Variable** | **HR (95% CI)** |
| **Demographics** |  |
| Age at index, years, mean ± SD | 1.01 (1.01, 1.01) |
| Male | 1.04 (1.01, 1.07) |
| American Indian or Alaska Native | 0.98 (0.83, 1.17) |
| Asian | 0.81 (0.75, 0.87) |
| Black or African American | 0.86 (0.81, 0.90) |
| Native Hawaiian or Other Pacific Islander | 0.92 (0.84, 1.01) |
| Other Race | 0.94 (0.87, 1.02) |
| White | 0.84 (0.80, 0.87) |
| Hispanic or Latino | 1.05 (1.00, 1.09) |
| **Comorbidities** |  |
| Non‑pressure chronic ulcer of lower limb (DFU) | 1.08 (1.02, 1.15) |
| Type 2 diabetes with diabetic kidney complications (DN) | 1.06 (1.01, 1.12) |
| Type 2 diabetes with peripheral angiopathy without gangrene | 1.07 (0.99, 1.14) |
| Type 2 diabetes with peripheral angiopathy with gangrene | 1.22 (1.05, 1.42) |
| Type 2 diabetes with polyneuropathy | 1.03 (0.99, 1.07) |
| Chronic kidney disease | 1.08 (1.03, 1.13) |
| End stage renal disease | 1.16 (1.07, 1.26) |
| Hypertensive diseases | 0.91 (0.88, 0.95) |
| Chronic lower respiratory diseases | 0.94 (0.90, 0.98) |
| Ischemic heart diseases | 0.97 (0.94, 1.01) |
| Heart failure | 1.15 (1.09, 1.21) |
| Atrial fibrillation and flutter | 0.98 (0.92, 1.04) |
| Cerebrovascular diseases | 1.03 (0.98, 1.08) |
| Arterial embolism and thrombosis | 1.13 (0.96, 1.33) |
| Atherosclerosis | 1.00 (0.94, 1.06) |
| Other peripheral vascular diseases | 1.03 (0.97, 1.09) |
| Other disorders of arteries and arterioles | 1.04 (0.96, 1.12) |
| Neoplasms | 0.97 (0.93, 1.00) |
| Persons with potential health hazards related to socioeconomic and psychosocial circumstances | 1.12 (1.04, 1.21) |
| Tobacco use | 1.06 (0.99, 1.15) |
| Fibrosis and cirrhosis of liver | 1.17 (1.05, 1.29) |
| **Medications** |  |
| Insulins and analogues | 1.06 (1.02, 1.10) |
| Blood glucose lowering drugs (including metformin) | 1.01 (0.97, 1.05) |
| Diuretics | 1.00 (0.96, 1.04) |
| Beta blocking agents | 1.04 (1.00, 1.08) |
| ACE inhibitors | 1.00 (0.97, 1.04) |
| Angiotensin II receptor blockers | 1.02 (0.98, 1.06) |
| Calcium channel blockers | 1.06 (1.02, 1.10) |
| Lipid modifying agents | 1.05 (1.01, 1.10) |
| Antithrombotic agents | 1.07 (1.02, 1.11) |
| **Laboratory values** |  |
| Haemoglobin A1c (%) | 0.87 (0.75, 1.01) |
| Body mass index (kg/m²) | 1.53 (1.36, 1.74) |
| Glomerular filtration rate (mL/min/1.73 m²) | 1.17 (0.87, 1.56) |
| Cholesterol (mg/dL) | 0.87 (0.78, 0.97) |
| Triglycerides (mg/dL) | 0.98 (0.88, 1.08) |
| **Healthcare Utilisation** |  |
| Ambulatory Visit | 1.06 (1.01, 1.11) |
| Emergency Department Visit | 1.08 (1.04, 1.12) |
| Inpatient Visit | 0.91 (0.88, 0.94) |
| Hazard ratios (HRs) and 95% confidence intervals (CIs) from a multivariable Cox proportional hazards model comparing patients with non-proliferative diabetic retinopathy (NPDR) who developed treatment-warranted dry eye disease (TW-DED) versus those who did not. The model adjusts for demographics, comorbidities (including diabetic foot ulcer [DFU] and diabetic nephropathy [DN]), medication use, laboratory values, and healthcare utilisation. An HR >1 indicates increased hazard of TW-DED progression. This analysis highlights the independent and combined contributions of DFU and DN to TW-DED risk, including racial and ethnic modifiers. | |
