# Supplementary material for: Racial and microvascular determinants of progression to treatment-warranted diabetic eye disease
Source: Eye (Lond). 2026 Apr 7;40(9):1371–7. doi: 10.1038/s41433-026-04417-0 (PMC13269473; doi:10.1038/s41433-026-04417-0)
Supplement: Supplementary file 2 — Supplemental Tables 7-8 [file 41433_2026_4417_MOESM2_ESM.docx]

| **Supplemental Table 7.** Race-Stratified Multivariable Cox Proportional Hazards Models on Progression to Treatment-Warranted Diabetic Eye Disease in Patients with Non-Proliferative Diabetic Retinopathy and Diabetic Neuropathy at Baseline | | | | | |
| --- | --- | --- | --- | --- | --- |
|  | **White** | **Hispanic** | **Black** | **Asian** | **Other** |
| **Variable** | **HR (95% CI)** | **HR (95% CI)** | **HR (95% CI)** | **HR (95% CI)** | **HR (95% CI)** |
| Effect of DN | 1.10 (1.04, 1.17) | 1.10 (0.97, 1.23) | 1.20 (1.07, 1.36) | 0.98 (0.76, 1.26) | 0.89 (0.70, 1.15) |
| **Demographics** |  |  |  |  |  |
| Age at index, years | 1.00 (1.00, 1.00) | 1.00 (1.00, 1.00) | 1.00 (1.00, 1.00) | 0.99 (0.99, 1.00) | 0.99 (0.99, 1.00) |
| Male | 1.00 (0.97, 1.03) | 1.07 (1.00, 1.14) | 0.95 (0.89, 1.01) | 1.12 (0.98, 1.29) | 1.03 (0.90, 1.17) |
| **Comorbidities** |  |  |  |  |  |
| Non‑pressure chronic ulcer of lower limb (DFU) | 1.22 (1.11, 1.34) | 1.39 (1.22, 1.60) | 1.27 (1.10, 1.47) | 1.17 (0.78, 1.73) | 1.32 (1.03, 1.70) |
| Type 2 diabetes with peripheral angiopathy without gangrene | 1.05 (0.96, 1.14) | 1.18 (1.01, 1.39) | 0.96 (0.81, 1.13) | 0.71 (0.44, 1.15) | 0.87 (0.65, 1.18) |
| Type 2 diabetes with peripheral angiopathy with gangrene | 1.25 (0.86, 1.81) | 0.85 (0.60, 1.20) | 1.31 (0.94, 1.81) | 0.35 (0.05, 2.58) | 2.18 (1.26, 3.78) |
| Type 2 diabetes with polyneuropathy | 1.10 (1.05, 1.16) | 1.14 (1.03, 1.25) | 1.12 (1.03, 1.23) | 0.98 (0.76, 1.27) | 1.10 (0.91, 1.32) |
| Chronic kidney disease | 1.01 (0.96, 1.06) | 1.13 (1.00, 1.27) | 0.94 (0.85, 1.03) | 1.12 (0.91, 1.38) | 0.98 (0.81, 1.19) |
| End stage renal disease | 1.15 (1.05, 1.26) | 1.27 (1.06, 1.53) | 1.17 (1.00, 1.38) | 0.83 (0.59, 1.16) | 1.06 (0.78, 1.45) |
| Hypertensive diseases | 0.90 (0.86, 0.93) | 0.87 (0.81, 0.95) | 0.91 (0.83, 0.99) | 0.91 (0.77, 1.08) | 0.89 (0.75, 1.05) |
| Chronic lower respiratory diseases | 0.82 (0.79, 0.86) | 0.82 (0.73, 0.91) | 0.83 (0.76, 0.90) | 0.88 (0.72, 1.07) | 0.82 (0.68, 0.98) |
| Ischemic heart diseases | 1.02 (0.98, 1.07) | 0.96 (0.86, 1.07) | 1.06 (0.96, 1.17) | 1.07 (0.88, 1.30) | 1.08 (0.90, 1.29) |
| Heart failure | 1.07 (1.01, 1.13) | 1.07 (0.94, 1.22) | 1.10 (0.99, 1.23) | 1.06 (0.80, 1.39) | 1.10 (0.87, 1.37) |
| Atrial fibrillation and flutter | 0.87 (0.81, 0.93) | 0.66 (0.53, 0.82) | 0.87 (0.74, 1.02) | 0.88 (0.62, 1.24) | 1.02 (0.80, 1.31) |
| Cerebrovascular diseases | 1.05 (0.99, 1.10) | 0.97 (0.86, 1.10) | 1.07 (0.97, 1.19) | 0.99 (0.79, 1.23) | 0.84 (0.67, 1.05) |
| Arterial embolism and thrombosis | 0.86 (0.69, 1.06) | 0.64 (0.40, 1.01) | 0.70 (0.47, 1.06) | 0.76 (0.31, 1.86) | 0.43 (0.18, 1.07) |
| Atherosclerosis | 0.90 (0.84, 0.98) | 0.91 (0.78, 1.06) | 0.81 (0.70, 0.94) | 1.10 (0.83, 1.47) | 1.13 (0.87, 1.49) |
| Other peripheral vascular diseases | 1.04 (0.97, 1.11) | 1.14 (0.98, 1.32) | 1.07 (0.93, 1.22) | 1.44 (1.06, 1.97) | 0.93 (0.72, 1.21) |
| Other disorders of arteries and arterioles | 1.00 (0.91, 1.10) | 0.90 (0.72, 1.12) | 1.13 (0.95, 1.35) | 1.10 (0.75, 1.62) | 0.94 (0.65, 1.36) |
| Neoplasms | 0.86 (0.83, 0.90) | 0.78 (0.72, 0.85) | 0.96 (0.88, 1.03) | 0.75 (0.63, 0.89) | 0.80 (0.68, 0.94) |
| Potential health hazards related to socioeconomic and psychosocial circumstances | 0.90 (0.82, 0.99) | 0.84 (0.71, 1.00) | 0.89 (0.75, 1.04) | 0.97 (0.63, 1.51) | 0.96 (0.63, 1.45) |
| Tobacco use | 0.81 (0.74, 0.89) | 0.72 (0.59, 0.89) | 0.95 (0.82, 1.10) | 0.75 (0.48, 1.20) | 0.85 (0.58, 1.24) |
| Fibrosis and cirrhosis of liver | 0.85 (0.76, 0.95) | 0.71 (0.58, 0.88) | 0.81 (0.63, 1.04) | 0.78 (0.45, 1.35) | 0.96 (0.58, 1.59) |
| **Medications** |  |  |  |  |  |
| Insulins and analogues | 1.24 (1.19, 1.29) | 1.22 (1.13, 1.33) | 1.30 (1.19, 1.41) | 1.28 (1.08, 1.53) | 1.25 (1.03, 1.53) |
| Blood glucose lowering drugs (including metformin) | 0.92 (0.88, 0.95) | 0.89 (0.81, 0.97) | 0.90 (0.83, 0.98) | 1.06 (0.88, 1.27) | 0.83 (0.69, 1.01) |
| Diuretics | 1.07 (1.02, 1.12) | 1.08 (0.99, 1.19) | 1.02 (0.94, 1.12) | 1.03 (0.85, 1.25) | 1.11 (0.90, 1.37) |
| Beta blocking agents | 1.05 (1.00, 1.09) | 1.16 (1.05, 1.27) | 1.02 (0.93, 1.12) | 0.76 (0.62, 0.92) | 0.93 (0.75, 1.16) |
| ACE inhibitors | 1.01 (0.97, 1.06) | 0.96 (0.88, 1.05) | 1.03 (0.95, 1.12) | 1.17 (0.98, 1.39) | 1.18 (0.96, 1.44) |
| Angiotensin II receptor blockers | 0.98 (0.94, 1.03) | 0.91 (0.82, 1.01) | 1.03 (0.94, 1.13) | 1.00 (0.83, 1.21) | 1.00 (0.80, 1.24) |
| Calcium channel blockers | 0.99 (0.94, 1.03) | 1.01 (0.92, 1.12) | 0.93 (0.85, 1.02) | 1.00 (0.83, 1.21) | 0.83 (0.67, 1.03) |
| Lipid modifying agents | 0.96 (0.92, 1.00) | 0.95 (0.87, 1.04) | 0.98 (0.89, 1.08) | 1.05 (0.86, 1.28) | 1.00 (0.81, 1.24) |
| Antithrombotic agents | 0.97 (0.93, 1.01) | 0.90 (0.82, 0.99) | 1.03 (0.94, 1.13) | 0.99 (0.82, 1.19) | 0.90 (0.73, 1.11) |
| **Laboratory values** |  |  |  |  |  |
| Haemoglobin A1c (%) | 0.75 (0.42, 1.36) | 1.20 (0.30, 4.81) | 0.56 (0.08, 3.98) | 0.69 (0.32, 1.22) | 0.80 (0.31, 2.95) |
| Body mass index (kg/m²) | 1.59 (1.40, 1.81) | 2.08 (1.43, 3.05) | 1.61 (1.36, 1.90) | 1.74 (0.93, 3.27) | 1.86 (1.04, 3.32) |
| Glomerular filtration rate (mL/min/1.73 m²) | 0.62 (0.46, 0.85) | 1.21 (0.73, 1.67) | 0.58 (0.34, 0.97) | 1.18 (0.16, 8.42) | 0.89 (0.65, 1.24) |
| Cholesterol (mg/dL) | 1.07 (0.96, 1.20) | 1.16 (0.79, 1.69) | 1.02 (0.84, 1.23) | 1.19 (0.58, 2.46) | 0.76 (0.28, 2.09) |
| Triglycerides (mg/dL) | 0.92 (0.83, 1.03) | 0.97 (0.67, 1.43) | 0.89 (0.74, 1.08) | 0.89 (0.43, 1.84) | 1.29 (0.47, 3.56) |
| **Healthcare Utilisation** |  |  |  |  |  |
| Ambulatory Visit | 0.90 (0.86, 0.94) | 0.93 (0.84, 1.03) | 1.07 (0.97, 1.19) | 0.74 (0.60, 0.91) | 0.83 (0.68, 1.01) |
| Emergency Department Visit | 1.01 (0.97, 1.04) | 0.96 (0.89, 1.03) | 0.98 (0.91, 1.06) | 1.08 (0.92, 1.26) | 0.93 (0.78, 1.10) |
| Inpatient Visit | 0.98 (0.94, 1.02) | 0.91 (0.84, 0.98) | 0.99 (0.91, 1.07) | 1.13 (0.95, 1.35) | 0.98 (0.81, 1.18) |
| Hazard ratios (HRs) with 95% confidence intervals (CI) from multivariable Cox proportional hazards models evaluating the association between diabetic foot ulcer (DFU) and progression to treatment-warranted diabetic eye disease (TW-DED) in patients with nonproliferative diabetic retinopathy (NPDR) and diabetic nephropathy (DN) at baseline. Analyses are stratified by race/ethnicity and adjust for demographic variables, comorbidities, medication use, laboratory values, and healthcare utilisation. An HR > 1 indicates increased TW-DED risk in patients with NPDR and DN. | | | | | |

| **Supplemental Table 8.** Race-Stratified Multivariable Cox Proportional Hazards Models on Progression to Treatment-Warranted Diabetic Eye Disease in Patients with Non-Proliferative Diabetic Retinopathy and DFU at Baseline | | | | | |
| --- | --- | --- | --- | --- | --- |
|  | **White** | **Hispanic** | **Black** | **Asian** | **Other** |
| **Variable** | **HR (95% CI)** | **HR (95% CI)** | **HR (95% CI)** | **HR (95% CI)** | **HR (95% CI)** |
| Effect of DFU | 1.21 (1.11, 1.33) | 1.42 (1.24, 1.62) | 1.25 (1.08, 1.44) | 1.08 (0.75, 1.57) | 1.27 (1.01, 1.61) |
| **Demographics** |  |  |  |  |  |
| Age at index, years | 1.00 (1.00, 1.00) | 1.00 (1.00, 1.00) | 1.00 (1.00, 1.00) | 1.00 (1.00, 1.00) | 1.00 (1.00, 1.00) |
| Male | 1.03 (0.98, 1.08) | 1.06 (0.99, 1.13) | 0.95 (0.89, 1.01) | 1.12 (0.98, 1.28) | 1.03 (0.90, 1.17) |
| **Comorbidities** |  |  |  |  |  |
| Type 2 diabetes with diabetic nephropathy (DN) | 1.10 (1.01, 1.21) | 1.13 (1.01, 1.28) | 1.24 (1.10, 1.41) | 1.02 (0.79, 1.33) | 0.97 (0.75, 1.26) |
| Type 2 diabetes with peripheral angiopathy without gangrene | 1.08 (0.96, 1.20) | 1.18 (1.01, 1.39) | 0.96 (0.81, 1.13) | 0.72 (0.44, 1.16) | 0.87 (0.64, 1.18) |
| Type 2 diabetes with peripheral angiopathy with gangrene | 1.08 (0.83, 1.40) | 0.84 (0.59, 1.18) | 1.31 (0.95, 1.82) | 0.37 (0.05, 2.71) | 2.25 (1.30, 3.90) |
| Type 2 diabetes with polyneuropathy | 1.07 (1.00, 1.14) | 1.13 (1.03, 1.24) | 1.13 (1.03, 1.24) | 0.95 (0.73, 1.23) | 1.11 (0.92, 1.33) |
| Chronic kidney disease | 1.04 (0.97, 1.12) | 1.12 (0.99, 1.25) | 0.93 (0.84, 1.03) | 1.12 (0.91, 1.37) | 0.97 (0.80, 1.18) |
| End stage renal disease | 1.07 (0.92, 1.26) | 1.27 (1.06, 1.53) | 1.18 (1.01, 1.38) | 0.82 (0.59, 1.15) | 1.05 (0.77, 1.43) |
| Hypertensive diseases | 0.94 (0.89, 1.01) | 0.87 (0.80, 0.94) | 0.90 (0.83, 0.99) | 0.91 (0.76, 1.08) | 0.90 (0.76, 1.07) |
| Chronic lower respiratory diseases | 0.83 (0.78, 0.89) | 0.82 (0.73, 0.91) | 0.83 (0.76, 0.91) | 0.87 (0.72, 1.06) | 0.82 (0.68, 0.98) |
| Ischemic heart diseases | 1.00 (0.94, 1.07) | 0.97 (0.87, 1.08) | 1.06 (0.96, 1.16) | 1.07 (0.88, 1.31) | 1.06 (0.89, 1.27) |
| Heart failure | 0.98 (0.90, 1.07) | 1.08 (0.94, 1.23) | 1.09 (0.98, 1.22) | 1.07 (0.81, 1.40) | 1.07 (0.86, 1.34) |
| Atrial fibrillation and flutter | 0.87 (0.80, 0.96) | 0.67 (0.54, 0.83) | 0.86 (0.73, 1.01) | 0.88 (0.62, 1.24) | 0.99 (0.77, 1.28) |
| Cerebrovascular diseases | 1.07 (1.00, 1.16) | 0.97 (0.86, 1.09) | 1.08 (0.97, 1.20) | 0.98 (0.78, 1.22) | 0.85 (0.68, 1.06) |
| Arterial embolism and thrombosis | 1.12 (0.90, 1.41) | 0.64 (0.41, 1.00) | 0.70 (0.46, 1.05) | 0.75 (0.31, 1.85) | 0.44 (0.18, 1.07) |
| Atherosclerosis | 0.91 (0.82, 1.00) | 0.91 (0.78, 1.06) | 0.82 (0.71, 0.95) | 1.13 (0.85, 1.50) | 1.13 (0.86, 1.48) |
| Other peripheral vascular diseases | 0.99 (0.90, 1.08) | 1.14 (0.99, 1.32) | 1.05 (0.92, 1.21) | 1.45 (1.06, 1.98) | 0.91 (0.70, 1.19) |
| Other disorders of arteries and arterioles | 1.02 (0.90, 1.16) | 0.92 (0.73, 1.14) | 1.12 (0.94, 1.34) | 1.11 (0.75, 1.63) | 0.98 (0.68, 1.41) |
| Neoplasms | 0.88 (0.83, 0.93) | 0.78 (0.72, 0.85) | 0.95 (0.88, 1.03) | 0.75 (0.63, 0.89) | 0.80 (0.68, 0.94) |
| Potential health hazards related to socioeconomic and psychosocial circumstances | 0.99 (0.86, 1.13) | 0.82 (0.69, 0.98) | 0.87 (0.74, 1.02) | 0.94 (0.60, 1.48) | 0.93 (0.61, 1.41) |
| Tobacco use | 0.83 (0.74, 0.94) | 0.76 (0.62, 0.93) | 0.95 (0.82, 1.11) | 0.76 (0.48, 1.20) | 0.85 (0.58, 1.25) |
| Fibrosis and cirrhosis of liver | 0.99 (0.85, 1.15) | 0.73 (0.59, 0.90) | 0.81 (0.63, 1.04) | 0.77 (0.44, 1.34) | 1.01 (0.62, 1.65) |
| **Medications** |  |  |  |  |  |
| Insulins and analogues | 1.24 (1.16, 1.31) | 1.23 (1.13, 1.33) | 1.30 (1.19, 1.41) | 1.29 (1.08, 1.53) | 1.24 (1.02, 1.52) |
| Blood glucose lowering drugs (including metformin) | 0.94 (0.88, 1.00) | 0.89 (0.82, 0.97) | 0.90 (0.83, 0.98) | 1.06 (0.88, 1.28) | 0.83 (0.68, 1.00) |
| Diuretics | 1.02 (0.95, 1.08) | 1.08 (0.99, 1.18) | 1.02 (0.93, 1.12) | 1.04 (0.86, 1.26) | 1.11 (0.90, 1.37) |
| Beta blocking agents | 1.05 (0.98, 1.12) | 1.14 (1.04, 1.25) | 1.02 (0.93, 1.12) | 0.76 (0.63, 0.92) | 0.94 (0.75, 1.16) |
| ACE inhibitors | 1.04 (0.98, 1.11) | 0.98 (0.89, 1.06) | 1.03 (0.94, 1.12) | 1.15 (0.97, 1.37) | 1.18 (0.96, 1.44) |
| Angiotensin II receptor blockers | 0.98 (0.91, 1.05) | 0.92 (0.83, 1.03) | 1.03 (0.94, 1.13) | 1.00 (0.83, 1.20) | 1.00 (0.80, 1.24) |
| Calcium channel blockers | 1.02 (0.95, 1.09) | 1.01 (0.92, 1.12) | 0.93 (0.86, 1.02) | 1.00 (0.83, 1.20) | 0.83 (0.67, 1.03) |
| Lipid modifying agents | 0.94 (0.87, 1.00) | 0.95 (0.87, 1.04) | 0.98 (0.89, 1.08) | 1.06 (0.87, 1.29) | 1.01 (0.82, 1.25) |
| Antithrombotic agents | 0.99 (0.92, 1.06) | 0.90 (0.82, 0.98) | 1.04 (0.95, 1.14) | 0.99 (0.82, 1.20) | 0.91 (0.74, 1.12) |
| **Laboratory values** |  |  |  |  |  |
| Haemoglobin A1c (%) | 0.78 (0.42, 1.45) | 1.21 (0.30, 4.85) | 0.56 (0.08, 3.96) | 1.03 (0.83, 1.43) | 0.64 (0.24, 1.58) |
| Body mass index (kg/m²) | 1.17 (0.91, 1.50) | 2.11 (1.44, 3.09) | 1.59 (1.34, 1.88) | 1.74 (0.92, 3.27) | 1.87 (1.05, 3.33) |
| Glomerular filtration rate (mL/min/1.73 m²) | 0.61 (0.43, 0.89) | 1.56 (0.51, 2.64) | 0.57 (0.34, 0.97) | 1.17 (0.16, 8.35) | 0.74 (0.42, 1.36) |
| Cholesterol (mg/dL) | 1.07 (0.92, 1.25) | 1.16 (0.79, 1.71) | 1.01 (0.84, 1.22) | 1.18 (0.57, 2.44) | 0.76 (0.28, 2.09) |
| Triglycerides (mg/dL) | 0.89 (0.76, 1.03) | 0.96 (0.66, 1.41) | 0.89 (0.74, 1.08) | 0.89 (0.43, 1.84) | 1.28 (0.46, 3.54) |
| **Healthcare Utilisation** |  |  |  |  |  |
| Ambulatory Visit | 0.95 (0.88, 1.02) | 0.93 (0.84, 1.02) | 1.08 (0.97, 1.20) | 0.74 (0.60, 0.92) | 0.84 (0.69, 1.02) |
| Emergency Department Visit | 1.05 (0.99, 1.11) | 0.95 (0.88, 1.03) | 0.98 (0.91, 1.06) | 1.07 (0.91, 1.25) | 0.93 (0.78, 1.11) |
| Inpatient Visit | 1.02 (0.96, 1.08) | 0.91 (0.84, 0.98) | 0.99 (0.92, 1.08) | 1.14 (0.96, 1.36) | 0.97 (0.80, 1.17) |
| Hazard ratios (HRs) with 95% confidence intervals (CI) from multivariable Cox proportional hazards models evaluating the association between diabetic nephropathy (DN) and progression to treatment-warranted diabetic eye disease (TW-DED) in patients with nonproliferative diabetic retinopathy (NPDR) and diabetic foot ulcer (DFU) at baseline. Analyses are stratified by race/ethnicity and adjust for demographic variables, comorbidities, medication use, laboratory values, and healthcare utilisation. An HR > 1 indicates increased TW-DED risk in patients with NPDR and DFU. | | | | | |
